# Comparison of Jump-Landing Biomechanics during and after Fatigue: Investigation on the Optimal Timing of Screenings under Fatigue

**DOI:** 10.5114/jhk/200421

**Published:** 2025-07-21

**Authors:** Stefan Vermeulen, Camilla De Bleecker, Valentien Spanhove, Veerle Segers, Tine Willems, Philip Roosen, Roel De Ridder, Jos Vanrenterghem

**Affiliations:** 1 Department of Rehabilitation Sciences, Ghent University, Ghent, Belgium.; 2 Department of Rehabilitation Sciences, Catholic University of Leuven, Leuven, Belgium.; 3 Department of Movement and Sports Sciences, Ghent University, Ghent, Belgium.

**Keywords:** screening, injury prevention, exertion, stop-jump

## Abstract

Fatigue has been considered a risk factor for sports injuries, modulating full-body jump-landing biomechanics. Biomechanical assessments of jump-landing manoeuvres are typically performed before and after short-term fatigue protocols, but changes during the protocol are often neglected. Therefore, this study investigated spike jump-landing strategy alterations during and following a short-term fatigue protocol in volleyball. Forty-three healthy, adult, male volleyball players participated in this study. Three-dimensional full-body kinematics were collected when performing spike jump-landings before, during and after a short-term fatigue protocol specific for volleyball. Full-body sagittal plane joint angles were calculated and analysed with curve analysis using one-way repeated measures ANOVA and post-hoc paired sample t-tests to investigate fatigue effects (p < 0.05). A significant main effect of fatigue was found for all kinematic variables (p = 0.015−0.041). More specifically, more pelvis-trunk flexion and less hip, knee, and ankle (dorsi-) flexion were observed during and after the protocol compared to baseline (p = 0.001−0.003). Moreover, less hip and knee flexion was observed during the protocol compared to after fatigue (p = 0.001−0.005). In conclusion, significant kinematic changes were found with fatigue, and these were somehow more pronounced during fatigue, possibly due to decreased attention towards the jump-landing task execution. A decision tree was provided to help researchers, coaches and/or clinicians in determining whether screenings should be better performed during or after fatigue, based on practical considerations.

## Introduction

Recently, fatigue has been more and more explicitly scrutinized as a candidate risk factor for injuries in sports with repetitive bouts of jump-landing tasks (e.g., volleyball, basketball) (Cieśla et al., 2015; Edwards et al., 2014; Lewis, 2018; Vermeulen et al., 2023a). In football and rugby, a high proportion of injuries occurs during late game stages or in the latter stages of the season, which possibly reflects the impact of fatigue on acute or overuse injury risk, respectively (Ekstrand et al., 2011; Gabbett, 2000; Hawkins et al., 2001; Price et al., 2004). Although the underlying mechanism for fatigue influencing injury risk is still unclear, fatigue at least negatively affects other risk factors for lower extremity injuries such as impaired neuromuscular control (Verschueren et al., 2020). Moreover, fatigue may alter jump-landing strategies in various ways, which may subsequently increase lower extremity injury risk (Barber-Westin and Noyes, 2017; Benjaminse et al., 2019; Harčarik et al., 2024; Santamaria and Webster, 2010; Vermeulen et al., 2023b).

Fatigue protocols are often included in biomechanical assessments to estimate lower extremity injury risk (Barber-Westin and Noyes, 2017; Benjaminse et al., 2019; Santamaria and Webster, 2010; Vermeulen et al., 2023b). Most biomechanical studies capture jump-landing tasks before and after short-term fatigue protocols, but this approach neglects changes occurring during the protocol itself. Evaluating biomechanical changes during fatiguing exercise may increase ecological validity by considering sports-specific fatigued conditions. This approach may reveal subtle changes to jump-landing strategies, as for example seen during intense volleyball rallies or match progression (Schmitz et al., 2014; Sheppard et al., 2007). Assessments during fatigue may also shift athletes’ main attention from the execution of the jump-landing task towards successfully completing the fatigue protocol, similar to real-time game scenarios. Likewise, amplified detrimental movement strategies have been observed when adding game-like situations to biomechanical assessments (Di Paolo et al., 2023).

To date, no studies have explicitly assessed biomechanical changes of sports-specific jump-landings during fatigue in volleyball or basketball. Three studies have compared alterations before and after fatigue protocols in these sports, finding reduced hip and/or knee flexion, but neglected changes occurring during the protocol itself (Edwards et al., 2024; Lin et al., 2021; Vermeulen et al., 2023a). Therefore, it remains unclear if similar strategies are also observed during fatigue and, if so, whether these changes are more or less pronounced compared to after fatigue. Identifying and comparing kinematic changes during and after fatigue will provide more insight into the optimal timing for future screenings under fatigue.

The purpose of this study was to assess and compare full-body kinematic changes to the most challenging jump-landing activity in volleyball, namely the horizontal landing/push-off phase preceding the spike jump, during a short-term sports-specific fatigue protocol and following the completion of the protocol. We hypothesized that kinematic changes, such as less hip and knee flexion, would occur with fatigue and that these might be more pronounced during fatigue than afterwards due to the addition of a sports-specific fatigued environment.

## Methods

### Participants

This study was registered at ClinicalTrials.gov (ID = NCT05161273) and approved by the Ethics Committee of the Ghent University Hospital, Ghent, Belgium. Additionally, written informed consent was obtained from each participant. For inclusion, participants had to meet the following criteria: (1) male competitive volleyball players, (2) at least 18 years old, (3) no current lower extremity injuries or in the past 3 months, and (4) no history of surgery to the lower extremities. This study was part of a larger prospective cohort study examining fatigue-induced biomechanical predictors for patellar tendinopathy in volleyball. Therefore, a convenience sample of 105 volleyball players was available to recruit from, of which 43 participants met the selection criteria for the present study (age: 22.8 ± 4.0 years; body mass: 79.5 ± 10.6 kg; body height: 184.0 ± 7.5 cm; body mass index: 23.5 ± 3.0 kg/m^2^; volleyball experience: 10.9 ± 6.1 years; volleyball participation: 6.7 ± 1.9 hours/week; player position: 10 outside hitters, 7 setters, 4 opposites, 11 middle blockers and 11 liberos; competition level (increasing difficulty): 12 provincial level, 23 regional level and 8 national level).

### Procedures

The test session started with a 10-min dynamic warm-up consisting of familiarization with the fatigue protocol without inducing any noticeable fatigue (Vermeulen et al., 2023a). Then, baseline (non-fatigued) spike jump-landing biomechanics were collected. When performing the spike jumps, participants ran from a self-selected distance towards a volleyball net (attached at a standardized height of 2.43 m), landed with both feet and took off vertically towards an imaginary ball positioned just above the net (Vermeulen et al., 2023a). Participants performed five baseline spike jump-landings, and two practice trials were allowed for familiarization. Next, volleyball players executed a high-intensity, intermittent exercise protocol (HIIP-5) to induce fatigue (Vermeulen et al., 2024). The HIIP-5 included a series of five circuits with volleyball-specific activities, each followed by 30 s of rest. The exact procedure of the HIIP-5 had been described in detail elsewhere (Vermeulen et al., 2024). One spike jump was performed immediately at the end of each circuit during the HIIP-5 as an integral part of the HIIP-5. No additional attempts were allowed after each circuit not to compromise the continuity of the HIIP-5. After completion of the fatigue protocol (i.e., after a rest interval of at least 30 s), participants again performed five (fatigued) spike jump-landings.

### Data Collection

Kinematic data were collected in a 4.5-m high laboratory setting (controlled for standard temperature) using 12 opto-electronic cameras (Qualisys, Oqus 3+ cameras, Gothenburg, Sweden, 300 Hz). Two force plates (Advanced Mechanical Technology, Inc., AMTI BP4602070RS-2K and AMTI BP12001200-4K-XS, Watertown, MA, USA, 1200 Hz) were embedded in front of the volleyball net (Huck, Koekelare, Belgium) for the validation of the event detection (cfr., ‘Data Analysis’). The same researcher (with a minimum of 5 years of physiotherapeutic experience and 2 years of academic background) placed retroreflective markers (12.5 mm, Qualisys, Gothenburg, Sweden) on the participants’ skin according to the Liverpool John Moores University biomechanical model (LJMU model) (De Bleecker et al., 2024; Malfait et al., 2014). This eight-segment model defines the trunk, the pelvis, upper legs, lower legs, and feet (Malfait et al., 2014).

To monitor exertion, the heart rate (HR, % of the theoretical maximum, i.e., 220 − age), rating of perceived exertion (RPE) scores and spike jump height were registered before, during and after the HIIP-5. The HR was monitored using a Polar heart rate system (Polar Electro, Inc., FT1 and FT2 polar watch, T31 chest strap, Helsinki, Finland) and can be considered a physiological marker for cardiovascular stress induced by the protocol (Stojanović et al., 2018). Perceived physical effort was measured by means of a subjective RPE-score for breathlessness (RPE-B) and legs (RPE-L) on a 20-point Borg scale (Borg et al., 2010). Physical performance was evaluated by changes in spike jump height (cfr., ‘Data Analysis’) to estimate muscular fatigue (Bossuyt et al., 2016).

### Data Analysis

Kinematic data were processed in Qualisys (Qualisys Track Manager, version 2020.2, Gothenburg, Sweden) and subsequently in Visual 3D software (C-motion, version 2021.11.3, Germantown, MD, USA). Kinematic data were filtered using a fourth order Butterworth filter at 20 Hz. Sagittal plane joint angles of the leading leg were calculated for the pelvis-trunk, hip, knee, and ankle. The horizontal landing/push-off phase preceding the actual spike jump was selected since it involves a demanding stretch-shortening cycle with a large load at the knee joint (Vermeulen et al., 2023a). Initial contact was defined as the first moment when the proximal end of the foot segment reached its lowest vertical position to the ground, while take-off was defined as the moment when the velocity of the distal end of the foot exceeded 1 m/s. Validity of this marker-based procedure was first checked and confirmed against the conventional force-based method for event detection (i.e., using the vertical component of the ground reaction force with a threshold set at 25 N). Kinematic data during horizontal landing/push-off were normalized to 100%, which resulted in one time profile for each joint from each individual spike jump in between the circuits during the HIIP-5 and one average time profile for each joint from five spike jump trials before and after the HIIP-5. Spike jump height was calculated as the difference of the maximal vertical height of the pelvic segment during the flight phase compared to the pelvic height during the standing static trial (Vermeulen et al., 2023a). Ultimately, kinematic time profiles and spike jump height were exported for statistical analysis to investigate differences between PRE (before HIIP-5), DURING (immediately at the end of each circuit of the HIIP-5) and POST (at least 30 seconds after HIIP-5) conditions.

### Statistical Analysis

The statistical analysis of the kinematic time profiles was performed in Matlab (The MathWorks, Inc., version R2023a, Natick, MA, USA) using Statistical Parametric Mapping (SPM, www.spm1d.org). Indicators of exertion were analysed in the SPSS statistical software package (IBM Corp., version V.28, New York, NY, USA). For all discrete outcome variables (i.e., indicators of exertion), normality was first checked and confirmed with the Shapiro-Wilk test and corresponding normality plots. Thereafter, one-way repeated measures ANOVA was performed for all outcome variables to investigate any main effect of fatigue. Post-hoc paired sample *t*-tests were performed with a Bonferroni correction for the number of executed tests within each variable. Overall, the level of significance was set at *α* = 0.05. Partial eta squared (*np^2^*) and pointwise Cohen’s *d* effect sizes were calculated for the omnibus ANOVA and post-hoc tests, respectively (small: *np^2^ =* 0.01−0.06, *d* = 0.20−0.50; medium: *np^2^ =* 0.06−0.14, *d* = 0.50−0.80; large: *np^2^* > 0.14, *d* > 0.80) (Richardson, 2011; Sullivan and Feinn, 2012). An a priori sample size calculation using G*Power software (G*Power, version 3.1.9.2, Aichach, Germany) determined that at least 30 players were needed to achieve 80% statistical power, *α* error (after Bonferroni correction) of 0.005 and an effect size of 0.68. The used effect size was based on a previous study that found significant decreased hip and knee flexion during the horizontal landing/push-off phase of a stop-jump task after fatigue (Edwards et al., 2014).

## Results

### Indicators of Exertion

Regarding the indicators of exertion (Table 1), a main effect of fatigue was observed for all variables (*p* < 0.001, *np^2^* = 0.67–0.96). More specifically, the HR and RPE-scores increased and jump height decreased DURING (*p* < 0.001, *d* = 1.46–6.08) and POST (*p* < 0.001, *d* = 0.94–4.01) compared to the PRE condition. However, the HR and RPE-scores generally decreased and jump height increased POST compared to the DURING condition (*p* = 0.001–0.002, *d* = 0.50–3.77).

**Table 1:** Indicators of exertion before, during and after the fatigue protocol (mean ± SD).

Timing ↓	Variables →	HR (% max)	RPE-B (6–20)	RPE-L (6–20)	Spike jump height (cm)
PRE		55.1 ± 6.9 ^a, b^	7.8 ± 1.7 ^a, b^	8.2 ± 1.9 ^a, b^	61.5 ± 6.6 ^a, b^
DURING	Circuit 1	91.3 ± 3.6 ^a, c^	13.6 ± 1.7 ^a, c^	10.8 ± 2.2 ^a^	52.0 ± 7.5 ^a, c^
	Circuit 2	94.3 ± 3.0 ^a, c^	15.7 ± 2.0 ^a, c^	12.2 ± 2.3 ^a, c^	52.4 ± 6.6 ^a, c^
	Circuit 3	95.7 ± 2.5 ^a, c^	16.8 ± 1.9 ^a, c^	13.7 ± 2.5 ^a, c^	51.3 ± 7.6 ^a, c^
	Circuit 4	96.2 ± 3.1 ^a, c^	17.6 ± 1.9 ^a, c^	14.7 ± 2.7 ^a, c^	51.1 ± 7.3 ^a, c^
	Circuit 5	96.8 ± 3.1 ^a, c^	18.3 ± 1.6 ^a, c^	15.6 ± 2.7 ^a, c^	49.8 ± 7.1 ^a, c^
POST		75.8 ± 5.6 ^b, c^	11.9 ± 2.6 ^b, c^	10.8 ± 2.8 ^b, c^	58.4 ± 6.0 ^b, c^

HR = Heart rate; RPE-B = Rating of perceived exertion for breathlessness; RPE-L = Rating of perceived exertion for legs; ^a^ Denotes a significant difference DURING vs. PRE (p < 0.05); ^b^ Denotes a significant difference POST vs. PRE (p < 0.05); ^c^ Denotes a significant difference POST vs. DURING (p < 0.05)

### Kinematic Time Profiles

**Figure 1:**
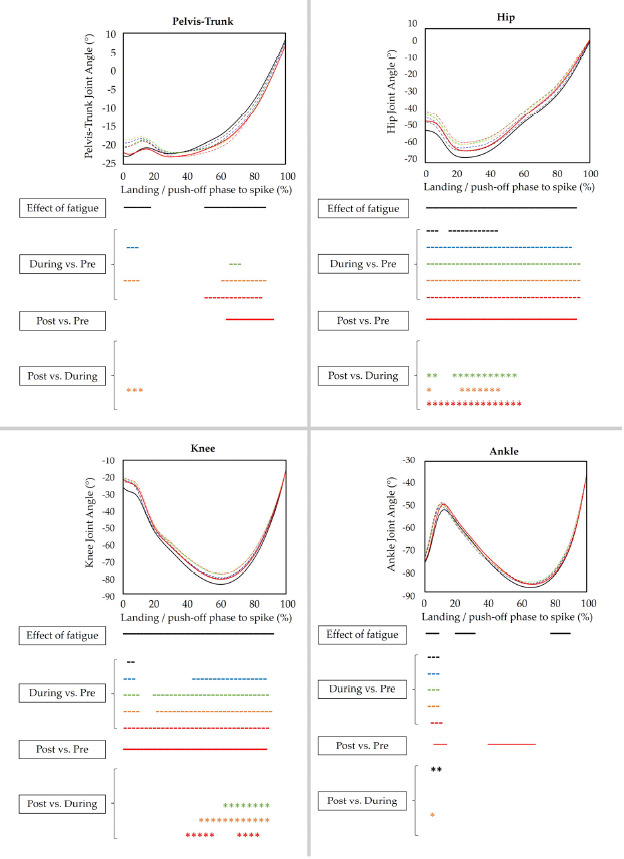
Full-body kinematics before, during and after the fatigue protocol. Upper, left: Pelvis-Trunk; Upper, right: Hip; Lower, left: Knee; Lower, right: Ankle. Negative values indicate (dorsi-) flexion. Mean curves are plotted for PRE, DURING and POST conditions (PRE = black, solid line, CIRCUIT 1 = black, dashed line, CIRCUIT 2 = blue, dashed line, CIRCUIT 3 = green, dashed line, CIRCUIT 4 = orange, dashed line, CIRCUIT 5 = red, dashed line, POST = red, solid line). Time frames of the kinematic curves that are significantly different between PRE, DURING and POST conditions are indicated underneath each figure (p < 0.05, CIRCUIT 1 vs. PRE = black, dashed line, CIRCUIT 2 vs. PRE = blue, dashed line, CIRCUIT 3 vs. PRE = green, dashed line, CIRCUIT 4 vs. PRE = orange, dashed line, CIRCUIT 5 vs. PRE = red, dashed line, POST vs. PRE = red, solid line, POST vs. CIRCUIT 1 = black asterisks, POST vs. CIRCUIT 2 = blue asterisks, POST vs. CIRCUIT 3 = green asterisks, POST vs. CIRCUIT 4 = orange asterisks, POST vs. CIRCUIT 5 = red asterisks)

The average and maximum time frames to complete the final kinematic assessments after the HIIP-5 were 3.5 min and 7 min, respectively. Regarding the kinematic variables (Figure 1), players generally performed the spike jump-landings with gradually more pelvis-trunk flexion (*p* = 0.001–0.003, *d* = 0.17−0.66), less hip flexion (*p* = 0.001–0.002, *d* = 1.67–3.40), less knee flexion (*p* = 0.001–0.003, *d* = 2.32–3.25) and less ankle dorsiflexion (*p* = 0.002, *d* = 0.52–1.34) DURING compared to the PRE condition. Similar strategies were observed in the POST compared to the PRE condition, in terms of more pelvis-trunk flexion (*p* = 0.001, *d* = 1.44), less hip flexion (*p* < 0.001, *d* = 3.32), less knee flexion (*p* < 0.001, *d* = 2.22) and less ankle dorsiflexion (*p* = 0.001–0.003, *d* = 1.89). However, hip and knee flexion again increased in the POST compared to the DURING condition (in particular when compared with circuit 3–5) (hip: *p* = 0.001–0.005, *d* = 1.82–2.51; knee: *p* < 0.001, *d* = 2.00–2.69). Few to no differences were observed for pelvis-trunk flexion and ankle dorsiflexion in the POST compared to the DURING condition.

## Discussion

The purpose of this study was to assess and compare full-body kinematic changes to spike jump-landing strategies before, during and after completion of a volleyball-specific fatigue protocol. In line with our hypothesis, we observed spike jump-landing strategy alterations with fatigue (i.e., more pelvis-trunk flexion and less hip, knee and ankle (dorsi-) flexion), which were more pronounced during the protocol compared to after the protocol completion.

This study observed more pelvis-trunk flexion and less hip, knee and ankle (dorsi-) flexion during spike jump-landings under fatigued conditions. Similar strategies have been already found during the horizontal landing/push-off phase of stop-jumps after fatigue (Edwards et al., 2014; Lin et al., 2021; Vermeulen et al., 2023a). Those studies speculated that less knee flexion might be a compensatory effect to prevent the knee from collapsing during landing in case of muscular fatigue in which players were unable to generate adequate knee extensor moments (Edwards et al., 2014). In line with this, more pelvis-trunk flexion may reduce and/or re-align impact forces acting on the stiffer distal joints and the fatigued knee extensor apparatus (Vermeulen et al., 2023b). Consequently, such kinematic alterations of the entire kinetic chain have already been associated with reduced patellar tendon loading in the fatigued state in populations that perform repeated bouts of demanding jump-landing tasks (e.g., volleyball, basketball) (Edwards et al., 2014; Vermeulen et al., 2023a). Hence, athletes who do not make appropriate kinematic compensatory adjustments when fatigued are assumed to be more prone for developing patellar tendinopathy, which is one of the most prevalent injuries in these sports (Edwards et al., 2014; Vermeulen et al., 2023a). These knee avoiding strategies, however, elicit substantial drops in jump performance (15−20% during and 5% after fatigue), which is disadvantageous and perhaps impossible in a competitive context (Vermeulen et al., 2023a). Furthermore, it is not known whether such strategies can be rather provocative for other links in the kinetic chain such as the thoraco-lumbar segments (that have a high injury incidence in these sports too), due to the higher demands that these strategies potentially place on them (Haddas et al., 2016; Kilic et al., 2017).

This study also showed that the observed alterations were more pronounced during the fatigue protocol compared to afterwards. Gradually more alterations were observed as the fatigue protocol progressed, followed by a (relatively) fast ‘normalisation’ of kinematic patterns after its completion, with hip and knee kinematics returning to those at the start of the protocol (i.e., circuit 1–2). This could be explained by a (relatively) quick recovery after our short-term fatigue protocol. Full muscle strength recovery has, for example, been reported within already 2–4 min after briefly locally fatiguing the hip and ankle muscles (Salavati et al., 2007). However, a recent study reported fatigue effects, in terms of reduced jump height and knee extensor muscle strength, lasting at least 30 min after the short-term HIIP-5 (Vermeulen et al., 2024). Similarly, reductions in hip and knee flexion were observed long (30–60 min) after discontinuation of sports-specific fatigue protocols (Bossuyt et al., 2016; Schmitz et al., 2014), suggesting that fast recovery might not be the only mechanism underlying our study findings. An alternative explanation could be an increased focus on the execution of the spike jump-landing task after fatigue. The fatigue protocol might have introduced an additional environmental factor, causing different neuromuscular control strategies due to distraction (Bolt et al., 2024), which are reflected in the smaller hip and knee flexion angles during the protocol compared to afterwards. Similarly, less knee flexion was also noted when distracting athletes by adding game-like situations to biomechanical assessments (Di Paolo et al., 2023). Given the potential for context-specific movement adaptations, similar conditions should be compared in future injury preventive screenings under fatigue (e.g., start vs. end protocol or pre vs. post protocol).

## Practical Implications

**Figure 2:**
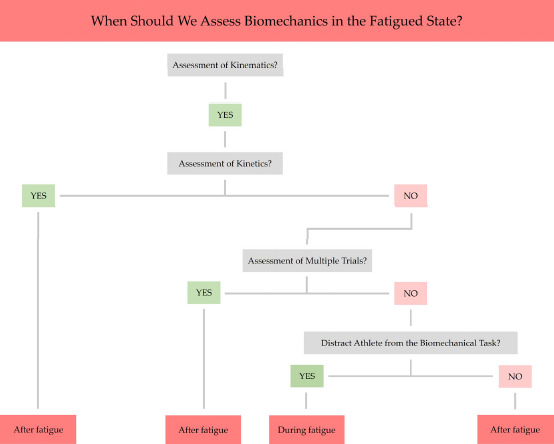
Decision tree for the optimal timing of biomechanical screenings under fatigue.

This study showed sufficient kinematic alterations under fatigue that have already been associated with sports-specific injury risk. This justifies continued implementation of fatigue protocols in future injury preventive screenings. Based on the results of this study, it appears that different multidisciplinary team members surrounding the athlete can select their own methods when deciding the best timing of these screenings (i.e., during or after fatigue). To assist researchers, coaches and/or clinicians in their decision-making, we provided an indicative decision tree for determining the optimal timing of screenings under fatigue (Figure 2). Researchers may, for example, opt to assess biomechanical alterations after fatigue to allow more time for re-applying markers that potentially fell off during the fatigue protocol and to record multiple trials of (synchronized force-)motion data in a standardized way. Coaches or supporting medical staff may choose to evaluate subtle changes to movement strategies during the fatigue protocol itself to better mimic gradual alterations and to distract the athlete as expected during real-time sports activity. Current advances in markerless motion capture technologies may soon make it possible to more easily undertake kinematic assessments as an integral part of (semi-standardized) on-field sports participation (Mauntel et al., 2021).

## Limitations

This study had some methodological considerations. First, we did not examine fatigue effects on kinetic variables since there were a lot of missing force data during execution of the HIIP-5 due to mis-hitting the force plates (i.e., only 9 out of 43 players fully touched the force plates for all assessments during the HIIP-5). Consequently, event detection of the horizontal landing/push-off phase was based on individual foot marker data, as described above. Second, only one spike jump-landing trial after each circuit during the HIIP-5 fatigue protocol was compared against the averages of five trials before or after the protocol, resulting in an unbalanced study design. To counteract this limitation, we a priori checked between-subjects variability through visual inspection of the width of all kinematic time profile’s standard deviation clouds and found no major differences. Third, we could not examine ‘true’ fatigue effects during the fatigue protocol itself as spike jump-landings were not assessed at the beginning of circuit 1 when there was no ‘fatiguing’ context. Fourth, participants were randomly scheduled for data collection at different times throughout the day, which may not have accounted for differences in mental and/or physical fatigue levels, possibly affecting (baseline) biomechanics and/or performance (Benjaminse et al., 2019; Van Cutsem et al., 2017). Fifth, only male participants were included in this study since gender differences may affect (fatigue-induced) jump-landing biomechanics (Kernozek et al., 2008). Hence, the study results cannot simply be extrapolated to the female population. Future studies could repeat this research design for other (phases within) frequently used tasks (e.g., block jump, sprinting, cutting) or for other sports or populations (e.g., women, basketball, soccer) to potentially generalize our findings.

## Conclusions

This study found more pelvis-trunk flexion and less hip, knee and ankle (dorsi-) flexion during spike jump-landings both during and after fatigue, which have been linked to altered injury risk. The kinematic changes were more pronounced during fatigue, most likely due to an external focus of attention. A decision tree was provided to help researchers, coaches and/or clinicians in determining whether screenings should be better performed during or after fatigue. This tree considers that assessments during fatigue better simulate sports-specific fatigued conditions, while post-fatigue screenings are more feasible to implement in a fully equipped laboratory setting. With advances in markerless motion capture technology, this seems promising for the future of on-field injury risk screening.
